# Functional Cortical Hubs in the Eyes-Closed Resting Human Brain from an Electrophysiological Perspective Using Magnetoencephalography

**DOI:** 10.1371/journal.pone.0068192

**Published:** 2013-07-09

**Authors:** Seung-Hyun Jin, Woorim Jeong, Jaeho Seol, Jiyeon Kwon, Chun Kee Chung

**Affiliations:** 1 MEG center, Department of Neurosurgery, Seoul National University Hospital, Seoul, Korea; 2 Department of Neurosurgery, Seoul National University Hospital, Seoul, Korea; 3 Neuroscience Research Institute, Seoul National University Medical Research Center, Seoul, Korea; 4 Department of Neurosurgery, Seoul National University College of Medicine, Seoul, Korea; 5 Seoul National University College of Medicine, Seoul, Korea; Beijing Normal University, Beijing, China

## Abstract

It is not clear whether specific brain areas act as hubs in the eyes-closed (EC) resting state, which is an unconstrained state free from any passive or active tasks. Here, we used electrophysiological magnetoencephalography (MEG) signals to study functional cortical hubs in 88 participants. We identified several multispectral cortical hubs. Although cortical hubs vary slightly with different applied measures and frequency bands, the most consistent hubs were observed in the medial and posterior cingulate cortex, the left dorsolateral superior frontal cortex, and the left pole of the middle temporal cortex. Hubs were characterized as connector nodes integrating EC resting state functional networks. Hubs in the gamma band were more likely to include midline structures. Our results confirm the existence of multispectral cortical cores in EC resting state functional networks based on MEG and imply the existence of optimized functional networks in the resting brain.

## Introduction

Recently, spontaneous brain activity in the resting state, defined as the absence of a task, has been investigated as a means to observe intrinsic brain activity. In functional magnetic resonance imaging (fMRI) studies, several resting state networks (RSNs) have been consistently observed across time and subjects [1]–[3], and the reliability of the intrinsic functional connectivity of the resting state has been confirmed [4]. Studies using electrophysiological modalities, including magnetoencephalography (MEG) [5]–[8] and electrocorticography [9], [10], have permitted the study of the dynamic integration of functional networks in the resting human brain with high temporal resolution.

Whole-brain functional networks can be investigated with MEG because of the wide coverage associated with this technique. Many MEG-based studies have focused on sensory-level functional networks in the resting state or during a task [11]–[14]. Recently, functional connectivity studies using MEG at the source space have been reported [5]–[8], [15]. Source-level connectivity analysis with MEG is recommended to reduce the well-known field spread effect [16], thus facilitating great advances in understanding RSNs from an electrophysiological perspective.

One of the advantages of investigating human brain networks is that it enables an examination of functional integration, one of the emergent properties of the brain. Human brain networks can be modeled as a number of nodes interconnected by a set of edges [17] and as a complex network of local and long-range connections [18], spurring efforts to identify “cortical core regions” or hubs in functional brain networks. Hubs ensure information integration in system-wide communication and contribute to brain economy as specialized “integrators” [19]. Among nodes composing the well-known default-mode network (DMN), the posterior cingulate cortex (PCIN) has been identified as a functional hub [8], and several other candidates have been suggested, depending on the frequency bands observed [15]. However, the greatest drawback of these previous studies involving spontaneous MEG activity is that the MEG signals were recorded in the eyes-open (EO) resting state with fixation. The resting state can be considered a behavioral state characterized by quiet repose with the eyes closed (EC) or EO with or without visual fixation [2]. However, the EO resting state with fixation can also be regarded as a passive fixation task. In fact, significantly higher functional connectivity and regional amplitude of low frequency fluctuations have been reported in both the EO with or without fixation conditions compared to the EC condition [20]. Moreover, we previously demonstrated that the EO resting state was associated with greater reproducibility in functional networks at sensor level than the EC resting state [14] because the EO resting state is associated with nonspecific or non-goal-directed visual information gathering and evaluation [20].

Here, unlike previous studies focusing on the EO resting state [8], [15], we focused on the EC resting state, which is an unconstrained state of free from any passive or active tasks, in order to investigate functional cortical cores. Therefore, the main aim of this study was to examine the functional cortical hubs in the EC resting state at different frequency bands using several known hub identification measures and to compare the results between subjects to determine whether there are cortical core regions across participants and frequency bands in a large population of 88 subjects.

To this end, we recorded MEG signals from 88 subjects in the EC resting state. We extracted the source activities at the 72 nodes (Table 1) covering the whole cortex on the basis of the automated anatomical labeling (AAL)-atlas [21]. Mutual information (MI), which quantifies the shared information between 2 time series based on information theory, was calculated to obtain the functional connectivity association matrix for each of 4 frequency bands corresponding to the classical EEG bands: theta (4–7 Hz), alpha (8–12 Hz), beta (13–30 Hz), and gamma (31–45 Hz). We characterized the functional cortical hubs by estimating graph-theory-based network measures, such as nodal degree (Dnodal), nodal efficiency (Enodal), normalized betweenness centrality (normBC), and modularity analysis, followed by hub classification. For Dnodal, Enodal, and normBC measures, a node larger than 2 SD was chosen as a hub after *z* score transformation. For modularity analysis, a hub node was classified by partitioning the network into modules and calculating the contribution of each node to inter- and intramodule connections represented in a z-P parameter plot (denoted as z-P).

**Table 1:** List of the anatomical regions of interest.

Anatomical description			Label
Central	Precentral gyrus		PRE
	Postcentral gyrus		POST
Frontal	Lateral Surface	Superior frontal gyrus, dorsolateral	F1
		Middle frontal gyrus	F2
		Inferior frontal gyrus, opercular part	F3OP
		Inferior frontal gyrus, triangular part	F3T
	Medial surface	Superior frontal gyrus, medial	F1M
		Supplementary motor area	SMA
		Paracentral lobule	PCL
	Orbital surface	Superior frontal gyrus, orbital part	F1O
		Superior frontal gyrus, medial orbital	F1MO
		Middle frontal gyrus, orbital part	F2O
		Inferior frontal gyrus, orbital part	F3O
		Gyrus rectus	GR
Temporal	Lateral Surface	Superior temporal gyrus	T1
		Heschl gyrus (transverse, BA41, 42)	HES
		Middle temporal gyrus	T2
		Inferior temporal gyrus	T3
Parietal	Lateral Surface	Superior parietal gyrus	P1
		Inferior parietal, but supramarginal and angular gyri	P2
		Angular gyrus	AG
		Supramarginal gyrus	SMG
	Medial Surface	Precuneus	PQ
Occipital	Lateral Surface	Superior occipital gyrus	O1
		Middle occipital gyrus	O2
		Inferior occipital gyrus	O3
	Medial and inferior surfaces	Cuneus	Q
		Calcarine fissure and surrounding cortex	V1
		Lingual gyrus	LING
		Fusiform gyrus	FUSI
Limbic	Temporal pole: superior temporal gyrus		T1P
	Temporal pole: middle temporal gyrus		T2P
	Anterior cingulate and paracingulate gyri		ACIN
	Median cingulate and paracingulate gyri		MCIN
	Posterior cingulate gyrus		PCIN
	Hippocampus		HIP

**Table 2:** Summary of most dominant hubs and hub identification measures in each frequency band.

	Dnodal				Enodal				normBC				z-P			
	θ	α	β	γ	θ	α	β	γ	θ	α	β	γ	θ	α	β	γ
F1_L	√	√	√	√	√	√	√	√	√	√	√	√	√	√	√	√
T2P_L	√	√	√	√	√	√	√	√	√	√	√	√	√		√	√
MCIN_L				√				√					√	√	√	√
MCIN_R				√				√					√	√		√
PCIN_L	√	√	√	√	√	√	√	√	√	√	√	√	√	√	√	√

A √ mark indicates that the node was identified as a hub.

## Materials and Methods

### Ethics Statement

The study protocol was approved by the local Institutional Review Board at Seoul National University Hospital (IRB no. H-0607-029-178). Written informed consent was given by all participants.

### Subjects

A total of 88 right-handed healthy subjects (mean ± SD: 24.28±4.03 years [range from 18 to 35], 54 males) voluntarily enrolled, and no participants had neurological problems. Handedness was tested using the Edinburgh Handedness Inventory [22].

### MEG Signal Acquisition and Preprocessing

We recorded MEG signals in the EC resting state (average duration, approximately 120 s). Magnetic fields (filter 0.1–200 Hz, 600 Hz sampling rate) were recorded inside a magnetically shielded room using a 306-channel whole-head MEG system (VectorView, Elekta Neuromag Oy, Finland). Head position relative to the sensor array was tracked with 4 additional head position indicator coils attached to the scalp. Signals were analog-filtered between 0.1 and 200 Hz and digitally sampled with a frequency of 600 Hz (when a different sampling frequency was used, a resampling process was applied to set the same frequency for all data). In addition to MEG, electrooculograms (EOG) and electrocardiograms (ECG) were simultaneously recorded. It should be noted that resting-state data were collected from participants who were engaged in several different studies; thus, the tasks that they participated in were different. However, the resting-state recordings in the eyes-closed state were conducted before the tasks.

As a preprocessing step, environmental and movement noise were removed with the temporal signal space separation (tSSS) method [23], which is a required and effective artifact removal preprocessing step for data recorded with the Elekta-MEG system [24]. Besides tSSS, no additional computerized corrections for eye-blinking and muscle movement were applied. Instead, the MEG signals having no excessive eye-blinking or eye-movement to minimize the artifacts were visually inspected, especially with the EOG signals. Epoching was done with Graph software (Elekta Neuromag Oy, Helsinki, Finland) after applying the tSSS method. We manually selected five 10-sec epochs each out of the continuous signal.

### Anatomical Segmentation and Extracting Source Waveforms from MEG Signals

The anatomical locations of 72 nodes (36 nodes in each hemisphere) are listed in Table 1, and corresponding MNI coordinates are shown in the Supplemental Information (Table S1). These 72 nodes were selected based on the AAL-atlas, which is used for anatomical parcellation of the brain [21]. We tried to include many nodes covered by the MEG recording among the AAL-atlas based nodes; thus, several sub cortical regions were excluded. The AAL-atlas has been used in studies that defined brain network nodes using fMRI [25]–[28].

Source waveforms were extracted from a set of 72 nodes covering the whole brain with BESA®2000 software (MEGIS Software GmbH, Germany). A template head implemented in BESA software was used when determining the source location instead of an individual MRI. MEG-MRI coregistration is possible using a template head under conditions that assure accurate digitization of individual head shape and with careful visual inspection [29]. We tried to follow this recommendation as much as possible. Note that it is not source imaging but reconstruction of source waveforms at 72 nodes in a simultaneous 72 dipole time-series extraction procedure.

### Estimation of Functional Connectivity

Various types of measures can be used for functional connectivity estimation, which include linear measures such as correlation and coherence, and nonlinear measures such as phase leg index and nonlinear synchronization [30]. Here, we choose MI which quantifies the shared information between two time series based on information theory. The main advantage of MI is that it can capture both linear and nonlinear relationships between time series.

From the extracted source waveform, we estimated MI values of the bandpass filtered waveforms to create an association matrix between the 72 nodes for each of 4 frequency bands corresponding to the classical EEG bands: theta (4–7 Hz), alpha (8–12 Hz), beta (13–30 Hz), and gamma (31–45 Hz). MI was calculated using the following equation:
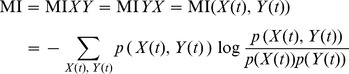
where 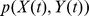 is the joint probability density function (PDF) between the 2 time series
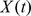
and 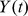. Thirty-two bins for the construction of the approximated PDF were adopted for 4096 samples like our previous study [31]. A corrective term was added when calculating MI to compensate for the effect of finite data and quantization on the PDF as proposed by Roulston [32]. MI matrices of each epoch and frequency band were calculated, and 5 MI matrices were averaged for the subsequent estimation of graph-theoretic measures [33]. MI is a relatively sensitive way to identify frequency-specific functional connectivity compared to cross-correlation, generalized synchronization, and phase synchronization [34]. To construct more accurate models of the complex brain networks, weighted graphs (MI matrices) were used for further graph-theoretic analyses. Because spurious correlations between MEG signals could occur because of the low spatial resolution of source-space MEG [8], [35] and the effect of spatial spread functions when multiple sources are estimated [36], pairs of nodes closer than 40 mm [37] were excluded in the present study. Grand averaged functional networks projected onto the cortex at each frequency are shown in Figure S1 (Supporting information).

### Nodal Network Metrics to Assess Nodal Centrality

The centrality of a node expresses its functional importance. Highly central nodes may serve as centers of information integration [38]. *N* is the set of all nodes, and *n* is the number of nodes. The total number of nodes was 72, corresponding to the number of source locations. The links between 2 nodes *i* and *j* are associated with the connection weight *w_ij_*. The weights were normalized by the maximum value of the MI matrix so as to produce 0≤ *w_ij_* ≤1 for all nodes. The shortest weighted path length of the path from node *i* to node *j*, the so called 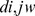, was calculated as 
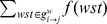
, where *f* is the inverse of the weight to length and 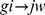
is the shortest weighted path between the 2 nodes, *i* and *j* [39].

To identify cortical hubs from the functional network, 3 centrality measures were employed: Dnodal, Enodal, and normBC. Dnodal indicates the total weight connected to a node, which refers to how strong the connection represented at a node is. Dnodal was calculated at each node *i* as 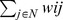 with the normalized connection weight *w_ij_*.

Enodal is regarded as a measure of communication efficiency [40] and is derived from the following equation.
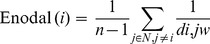



Betweenness centrality (BC) measures how often nodes occur on the shortest paths between other nodes [41], [42]. It is defined as the following equation:


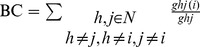
, where 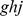 is the number of shortest paths between node *h* and *j* and
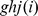
is the number of shortest paths between node *h* and *j* passing through *i*. BC was normalized by the mean value of BCs in a network [43], [44], and thus, we denoted it as normBC.

These analyses were performed with the Brain Connectivity Toolbox (http://www.brain-connectivity-toolbox.net/). When necessary, the scripts were modified. Each nodal network metric was standardized by conversion to *z* scores as follows:
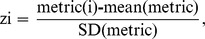
where ‘metric’ indicates each nodal network metric applied such as the Dnodal, Enodal, and normBC; metric (i) denotes the nodal network metric at node i. Mean (metric) is the mean nodal network metric across all nodes in the network, and SD (metric) is the standard deviation of the network. The conversion to *z* scores does cause the values in each subject map to be comparably scaled [41]. The nodes with *z* scores larger than 2 SD were chosen as a hub for each nodal network metric.

The conversion to *z* scores does cause the values in each subject map to be comparably scaled [41]. The nodes with *z* scores of larger than 2 SD were chosen as a hub for each nodal network metric.

### Community Detection with Modularity and Hub Classification

Modularity analysis was performed to identify hubs based on the network community structure. In accordance with previous studies [35], [39], the modularity of brain networks suggests that the nodes in any module will be more densely connected to each other than to nodes in other modules, as defined by.


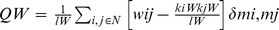
, where 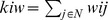 is a weighted degree of node *i* and 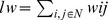 is a sum of all weights in the network.

Hub locations were classified from the modularity analysis. The within-module degree *z* score, which indicates how well-connected the node *i* is to other nodes within module, and the participation coefficient *PC*, which assesses the diversity of intermodular interconnections of individual nodes [35], [39], were used for hub classification as follows:
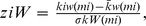
where *m_i_* is the module containing node *i*, 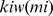 is the within-module degree of *i* (the number of links between *i* and all other nodes in *m_i_ )*, and 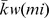
and 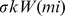 are the respective mean and standard deviation of the within-module *m_i_* degree distribution.



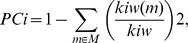
where *M* is the set of modules and 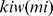 is the number of links between *i* and all nodes in module *m*.

With those measures, we adopted a conservative hub classification method using the z-P parameter space proposed by Guimera and Amaral [45]. According to the within-module degree, we classified nodes with *z* ≥2.5 as module hubs and nodes with *z* <2.5 as nonhubs. More specific roles of each node were characterized with the values of the participation coefficient [45]. Provincial (*PC* ≤0.30), connector (0.30<*PC* ≤0.75), and kinless (*PC* >0.75) hubs were classified at each condition and frequency band.

## Results

### Functional Cortical Hubs with High Degree

We first identified hubs by identifying nodes with a high degree of connectivity using Dnodal.

**Figure 1:**
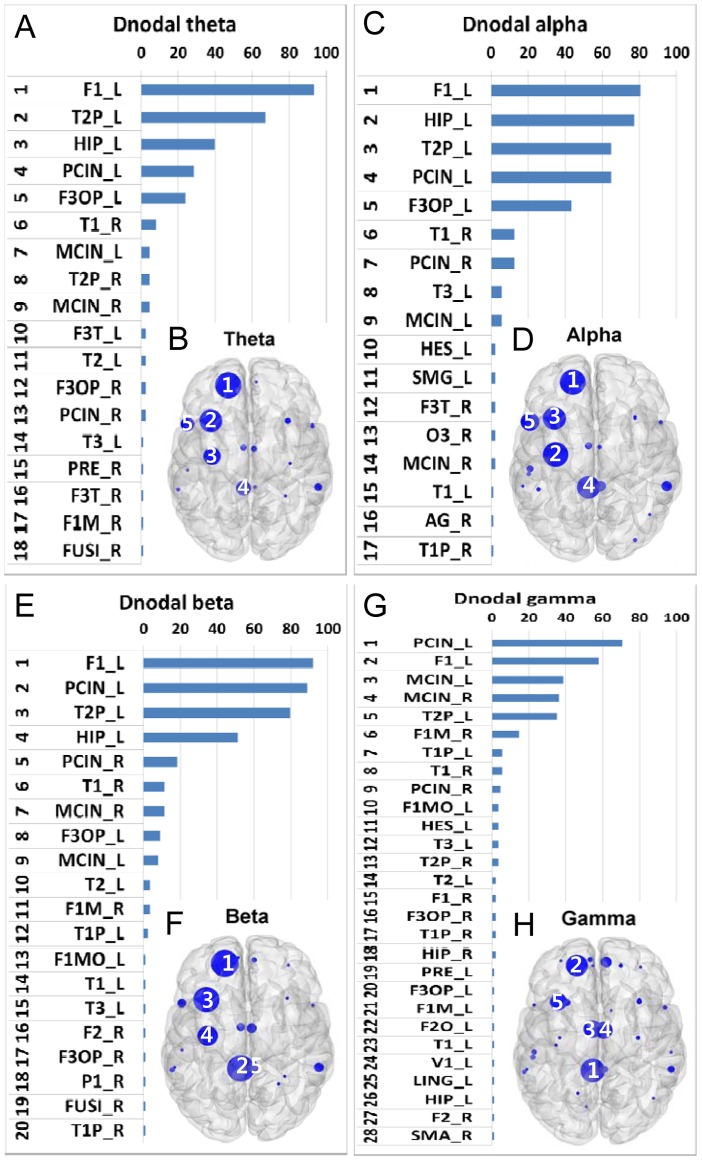
Hubs with high degree. Hubs based on the aggregated ranking percent of each node across 88 participants and their topological maps projected into a cortical surface at the theta (A and B), alpha (C and D), beta (E and F), and gamma (G and H) bands obtained from Dnodal estimation. The ranked distribution of aggregated ranking percent included only nonzero percent nodes, and the numbers in the topological maps denote the top 5 hub locations. Abbreviated notations for each node can be found in Table 1, and ‘_L’ and ‘_R’ denote the left and right hemisphere, respectively, at each node. The horizontal axes in A, C, E, and G indicate percentage (%).

Figure 1 shows hubs based on their aggregated ranking percent across 88 participants and their topological maps projected into a cortical surface at each frequency band. Overall, there are similarities in the hub locations from the theta to beta bands, although the percentage varies with the frequency bands. By contrast, the gamma band hubs were somewhat different from the hub distributions in the other frequency bands.

Specifically, in the theta band, the left dorsolateral superior frontal gyrus (F1_L), the left pole of the middle temporal gyrus (T2P_L), the left hippocampus (HIP_L), the left posterior cingulate gyrus (PCIN_L), and the left inferior frontal gyrus (F3OP_L) were identified as the top 5 hubs (Figure 1A and 1B). The most prominent hub was found at F1_L, where a hub was identified in 93% of the 88 subjects. In the alpha band, the top 5 hubs were located at the same locations as the theta band: F1_L, HIP_L, T2P_L, PCIN_L,, and F3OP_L. However, the number of subjects was different; approximately 81% of all subjects peaked at F1_L (Figure 1C and 1D). In the beta band, nodes at F1_L, PCIN_L, T2P_L, and HIP_L were again observed, and the right posterior cingulate gyrus (PCIN_R) appeared as a hub (Figure 1E and 1F). The most prominent hub location was also the same as in the theta and alpha bands (F1_L), with 92% of all subjects peaking at this location. The dominant role of F1_L was supported by its appearance as the top hub over the entire theta to beta frequency range (4–30 Hz). The commonly found hubs, such as the PCIN_L, T2P_L, and HIP_L might be important hubs over the theta to beta bands as well. In the gamma band, the bilateral median cingulate gyri (MCIN_L & MCIN_R) were among the top 5 hubs. Notably, hub locations in the gamma band were different from previous bands, but F1_L, T2P_L, and PCIN_L still remained in the top 5 nodes (Figure 1G and 1H). PCIN_L was the most prominent hub node in 71% of subjects.

### Functional Cortical Hubs with High Efficiency

Efficiency in brain networks, defined as the inverse of the harmonic mean of the shortest path length between one node and all other nodes, is regarded as a measure of the communication efficiency in the network [46]. Therefore, a hub with high efficiency can be regarded as the main location of information processing.

**Figure 2:**
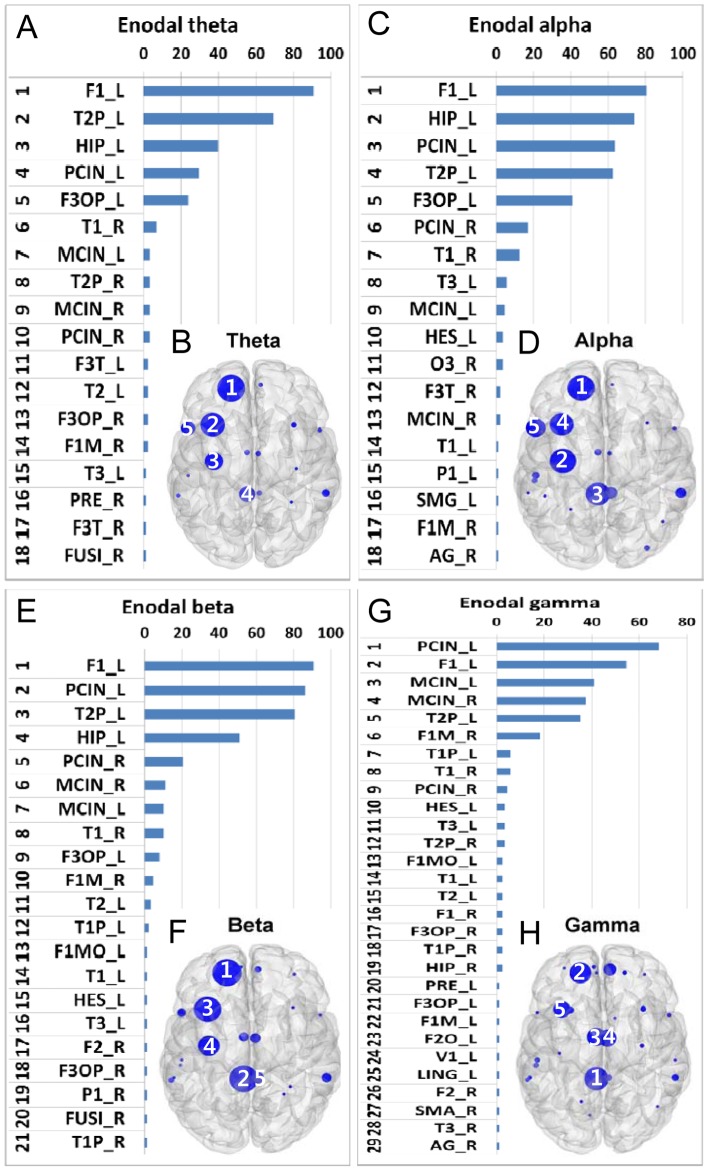
Hubs with high efficiency. Hubs based on the aggregated ranking percent of each node across 88 participants and their topological maps projected into a cortical surface at the theta (A and B), alpha (C and D), beta (E and F), and gamma (G and H) bands obtained from Enodal estimation. The ranked distribution of aggregated ranking percent included only nonzero percent nodes, and the numbers in the topological maps denote the top 5 hub locations. Abbreviated notations for each node can be found in Table 1, and ‘_L’ and ‘_R’ denote the left and right hemispheres, respectively, at each node. The horizontal axes in A, C, E, and G indicate percentage (%).

Ranked distributions of hubs based on the aggregate ranking percent across 88 subjects and their topological maps characterized by Enodal are shown in Figure 2. As in the Dnodal analysis, the hubs from the theta to beta bands were similar. By contrast, hubs in the gamma band network were distributed mainly over midline structures.

In the theta (Figure 2A and 2B) and alpha (Figure 2C and 2D) bands, F1_L, T2P_L, PCIN_L, HIP_L, and T2P_L were the top 5 hubs. In the beta band, the top 5 hubs were located at F1_L, PCIN_L, T2P_L, HIP_L, and PCIN_R. F1_L was again the most prominent hub and was found in 91% of subjects (Figure 2E and 2F). In the gamma band, cortical midline structures appeared as hubs, including the cingulate cortex, PCIN_L, MCIN_L, and MCIN_R. T2P_L was the major hub (Figure 2G and 2H). PCIN_L was identified as a hub in 60 subjects.

### Functional Cortical Hubs with High Centrality

Betweenness centrality is defined as the fraction of the shortest paths in the network that pass through a given node [39]. A region with high betweenness centrality represents a position that frequently becomes a shortcut between nodes within the network.

**Figure 3:**
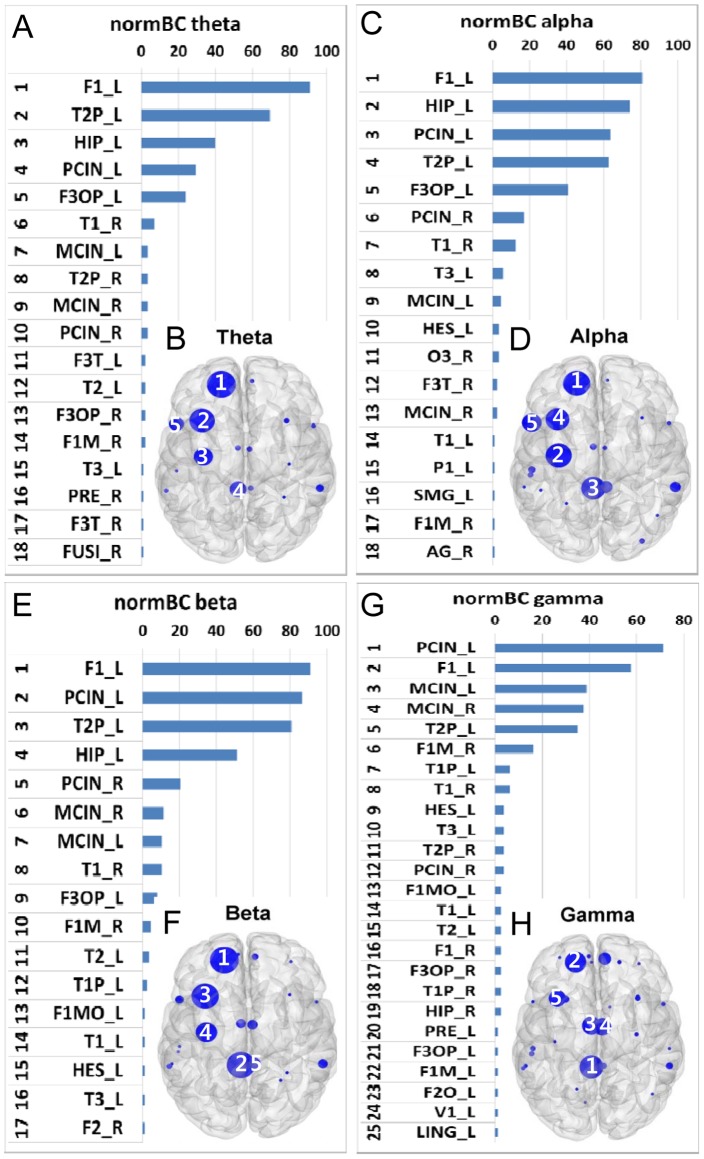
Hubs with high centrality. Hubs based on the aggregated ranking percent of each node across 88 participants and their topological maps projected into a cortical surface at the theta (A and B), alpha (C and D), beta (E and F), and gamma (G and H) bands obtained from normBC estimation. The ranked distribution of aggregated ranking percent included only nonzero percent nodes, and the numbers in the topological maps denote the top 5 hub locations. Abbreviated notations of each node can be found in Table 1, and ‘_L’ and ‘_R’ denote the left and right hemispheres, respectively, at each node. The horizontal axes in A, C, E, and G indicate percentage (%).

Ranked distributions of hubs based on aggregated ranking percent across all subjects and their topological maps characterized by normBC are shown in Figure 3.

In the theta band, F1_L, T2P_L, HIP_L, PCIN_L, and F3OP_L were the top 5 hubs in order, and F1_L was a hub in 80 of 88 subjects (91%), making it the most prominent hub (Figure 3A and 3B). In the alpha band, F1_L, HIP_L, PCIN_L, T2P_L, and F3OP_L were the top 5 hubs. The most prominent hub location was the same as in the theta band (F1_L) but occurred in a slightly low percentage of subjects, 81% (Figure 3C and 3D). In the beta band, F1_L, PCIN_L, T2P_L, HIP_L, and PCIN_R were the top 5 hubs (Figure 3E and 3F). In the gamma band, the top 5 hubs were PCIN_L, F1_L, MCIN_L, MCIN_R, and T2P_L (Figure 3G and 3H). The most prominent hub location was PCIN_L, which was identified in 71% of subjects. Again, the main hubs in the gamma band appeared to be located in the vicinity of midline structures.

### Functional Cortical Hubs with a High Degree and Centrality

Modularity analysis was also performed to identify hubs based on the network community structure. The modularity measure and the hub classification within a module [45] provide useful tools for understanding network structure [13], [35], [47]. We accepted a conservative hub classification criteria using the z-P with the within-module degree *z* score and the participation coefficient (*PC*) determining the universal role of a node within a module based on the method introduced by Guimera and Amaral [45]. Using this method, it is possible to derive hubs with high degree and centrality.

**Figure 4:**
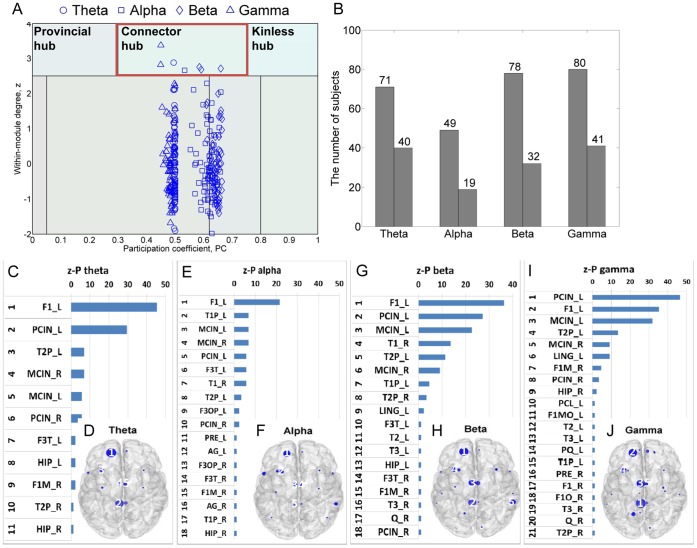
Functional cartography of a subject using the within-module *z* score and participation coefficient (*PC*) of each node, and hubs with high degree and centrality derived from modular structure. Seven hubs were characterized as connector hubs (red box) in this subject. Nodes were classified as hubs according to z-P cartography for all subjects (A). Because all subjects presented hubs, Figure 4B displays the number of subjects who had hubs (first bar and figure) and the number of subjects who had each predominant hub depending on frequency bands (second bar and figure). Hubs were characterized in 71, 49, 78, and 80 of 88 subjects depending on frequency bands, and among them, 40, 19, 32, and 41 subjects demonstrated each predominant hub at each frequency band. Hubs based on the aggregated ranking percent of each node across 88 participants and their topological maps projected into a cortical surface at the theta (C and D), alpha (E and F), beta (G and H), and gamma (I and J) bands obtained from the modularity-based hub identification method are shown. The ranked distribution of the aggregated ranking percent included only nonzero percent nodes, and the numbers in the topological maps denote the top 5 hub locations. Abbreviated notations for each node can be found in Table 1, and ‘_L’ and ‘_R’ denote the left and right hemispheres, respectively, at each node. The horizontal axes in A, C, E, and G indicate percentage (%), not corrected by the number of subjects who had hubs in Figure 4B.

Figure 4A displays the z-P of a subject, in which several hubs can be observed. Interestingly, all identified hubs across subjects were connector hubs that have high degree and centrality, but, by definition, their connections run between 2 or more modules. Overall, the number of subjects with identified hubs using the z-P method was less than previous hub identification measures, which may be due to the strict definition of a hub by a *z* score larger than 2.5 within a module. In fact, no hubs were identified in many subjects (Figure 4B).

In the theta band, the top 5 hubs were F1_L, PCIN_L, T2P_L, MCIN_R, and MCIN_L (Figure 4C and 4D). The most prominent hub was F1_L, identified in 45% of subjects. In the alpha band, the top 5 hubs were F1_L, the left superior temporal pole (T1P_L), MCIN_L, MCIN_R, and PCIN_L (Figure 4E and 4F). Even the most prominent hub (F1_L) was found in only 19 of 88 subjects (22%). In the beta band, F1_L, PCIN_L, MCIN_L, T1_R, and T2P_L appeared as hubs (Figure 4G and 4H). Again, the most prominent hub was F1_L, which was found in 36% of subjects. In the gamma band, PCIN_L, F1_L, MCIN_L, T2P_L, and MCIN_R were the top 5 hubs (Figure 4I and 4J). Among them, PCIN_L was found in the greatest number of subjects, approximately 47%. If we eliminated the subjects who had zero hubs, PICN_L was identified in all subjects (41/41).

### Consistent Hubs across Hub Identification Measures

**Figure 5:**
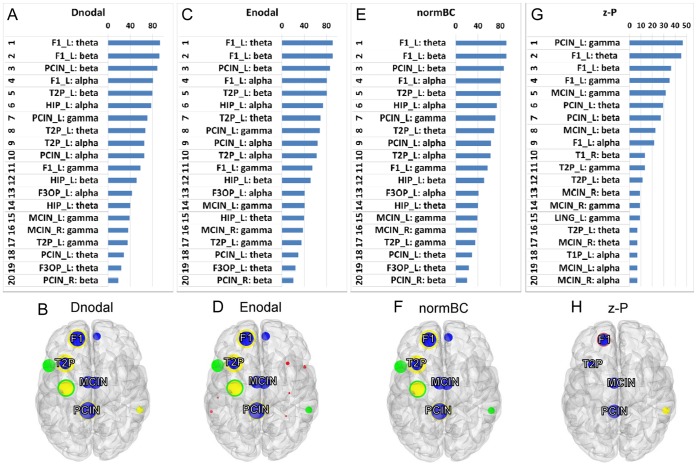
Ranked distribution of Dnodal (A, B), Enodal (C, D), normBC (E, F), and z-P (G, H). Shown are the top 20 hubs based on the aggregated ranking percent of each node across 88 participants and their topological maps projected into a cortical surface derived from Dnodal (A and B), Enodal (C and D), normBC (E and F), and z-P (G and H) measures irrespective of frequency bands. The most consistent hubs, F1, T2P, MCIN, and PCIN, are marked in each topological map. Horizontal axes in A, C, E, and G indicate percentage (%). The size of the filled circles is proportional to the corresponding percent, and the color indicates each frequency band (Theta: red, alpha: green, beta: yellow, gamma: blue).

The above analyses focused on identifying functional hubs in different frequency bands using multiple hub identification methods. To identify dominant hubs based on the hub identification measurements, we investigated the ranked distribution of all nodes irrespective of frequency bands. F1_L in the theta (Dnodal, Enodal and normBC) band was the most prominent hub (Figure 5A, 5C, and 5E). PCIN_L in the gamma band was the top hub in z-P (Figure 5A). The most consistent hubs across hub identification methods were PCIN_L, F1_L, MCIN, and T2P_L (Figure 5B, 5D, 5F, and 5H). As seen above, hubs in the gamma band were more likely to include midline structures such as the median and posterior cingulate regions.

F1_L appeared across all frequency bands and hub identification measures, meaning that this node is highly efficient, with high degree and centrality from the theta to beta bands. T2P_L and PCIN_L were characterized as hubs in almost all cases, across all frequency bands and methodologies. Although PCIN_R was seen in the beta band with high degree and efficiency, and in the beta band with high centrality, its contribution appeared to be minimal compared to PCIN_L. This suggests that F1_L, T2P_L, and PCIN_L are global hubs that act as connectors in functional brain networks in the EC resting state from the theta to beta bands. By contrast, the bilateral MCIN were identified as hubs in the gamma band with all measures.

## Discussion

Here, we demonstrated the existence of multispectral cortical hubs integrating the EC resting state functional networks by electrophysiological methods. Cortical hubs varied slightly with different hub identification measures and frequency bands. The most consistent hubs across hub identification measures were observed in the medial and PCIN and the left dorsolateral superior frontal and middle temporal pole regions. They were characterized as connector hubs that play an important role in promoting information flow between otherwise segregated brain regions. In the gamma band, midline structures such as the median cingulate cortices were predominantly involved. The functional hubs that we identified could be the cores of the electrophysiological self, representing embedded self-referential or self-regulatory processes and the manifestation of optimized multispectral functional networks ready to react to unexpected external stimuli. A system of brain oscillators allows brain operations to be performed simultaneously at multiple temporal and spatial scales [48], [49]. Electrophysiological approaches such as those used in our study have a distinct advantage over fMRI, which measures hemodynamic responses and may be insufficient to capture complex brain systems.

### Functional Cortical Hubs in the EC Resting State

We identified several functional cortical hubs in the EC resting state, an unconstrained state free from any given passive task, based on MEG analysis of 88 participants. A close relationship between the RSNs identified by fMRI and the electrophysiological resting state obtained from MEG was confirmed previously [6]–[8], and frequency-specific hub candidates were suggested [15] in the EO resting state with fixation. However, the literature confirms that the network features in the EO condition are different from the EC condition, even though the physiological principles underlying these differences have yet to be established. In fact, in the EO with or without fixation condition, significantly higher functional connectivity [50] and a robust estimation of functional connectivity compared with the EC condition were demonstrated [20]. The EO state also exhibits greater reproducibility compared to the EC state [14]. In the present study, to overcome the lack of robustness in the EC resting state, we analyzed MEG signals obtained from a large population of 88 participants. Our results provide several missing pieces of information about spontaneous brain activity by confirming the existence of dominant functional cortical hubs in the EC resting state by electrophysiological methods.

Although our results included similarities and dissimilarities with previously reported structural and functional hubs, we presume that the hubs we identified are the manifestation of characteristic features of the EC resting state that differ from those of the EO resting state. In a previous study, global hubs in the EO resting state were identified in the medial temporal lobe in the theta band, in lateral parietal areas in the alpha to beta bands and in sensorimotor areas for higher frequencies [15]. By contrast, the hubs identified in our study were located mainly in the cingulate cortex (PCIN, MCIN), the left dorsolateral superior frontal cortex (F1_L), and the left middle temporal pole (T2P_L). Hubs appeared over almost all frequency bands, with the exception of the preference of MCIN for the gamma band. PCIN and F1_L largely overlapped with core regions identified by structural connectivity [38], [41], [51], [52], which implies a close relationship between functional hubs in the EC resting state and structural hub regions. In addition, PCIN was also identified as a hub in the EO resting state [8]. Even in the same EO resting state, the main hubs are not identical [8], [15], possibly due to differences in node selection, i.e., seed-based [8] versus the whole brain [15]. In fact, the seed-ROIs used by de Pasquale and colleagues [8] were selected based on the RSN components identified using fMRI, while more dense nodes covering the entire cortex were used by Hipp and colleagues [15]. Since we do not know about the physiological principles underlying differences between the EC and EO resting states as of yet, the investigation of the physiological principles in the EC and EO resting states would be the next research topic.

### Potential Functional Roles of Hubs and their Contribution to the Resting State Brain Network

Because spontaneous brain activity reflects endogenous network activity in the brain, which is metabolically costly [53], and cortical connectivity plays a crucial role in shaping resting state neural dynamics [19], cortical hubs in the resting state may have functional significance. One of the most important goals of this study was to identify hubs and their characteristics. Thus, it is interesting that the hubs we identified are connector hubs, which reflect the hub’s contribution to facilitating functional integration and their important role in system-wide information integration. According to the definition, hub nodes can be naturally categorized into 3 different roles: provincial, connector, and kinless hubs [45]. Connector hubs are hubs with many links to most of the other modules. Because hub nodes have high values of within-module connectivity by definition, a connector hub node is a well-connected node within a module and is simultaneously responsible for many between-module connections.

PCIN stands out as a hub over almost all frequency bands and hub identification measures, indicating its global influence in maintaining resting state functional networks regardless of spectral characterization with high degree, centrality and efficiency. There is a large body of work on PCIN in the resting state, which is characterized by high metabolic activity [54]–[57], structural connectivity [38], [41], [51], [52], functional connectivity [8], [41], and even a developmental role [58]. The presence of PCIN as a hub is agreement with previous findings on PCIN as a cortical site that allows communication between different cortical modules previously described in a MEG study [8], and PCIN as extremely robust functional cores at rest that represent a convergence site for both intra- and inter-network interactions described in a recent fMRI study [59]. Of course, PCIN overlapped with the region constituting DMN [56], [60], which might support the role of DMN allowing the off-line internally focused processes [59].

F1_L was another dominant functional hub. Again, the dorsolateral superior frontal cortex is one of the components of the DMN [1], [59], [61]. As mentioned above, PCIN and F1_L may be associated with structural connectivity. In addition, dense long-range connections between the superior frontal cortex and PCIN are more prevalent in adults compared to children [58], which emphasizes an important developmental role of long-range connections between the F1_L and the PCIN regions. Our results suggest that F1_L and PCIN contribute to information integration within the cerebral cortex.

The temporal pole (BA = 38) has been positively correlated with PCIN during rest [62]. The temporal pole is one of the components involved in “mentalizing”, the ability to represent the mental state of both oneself and other people [63]. There is general agreement among many studies about the role of the temporal pole in the theory of mind [64], in line with the mentalizing concept. Thus, the appearance of T2P_L over almost all frequency bands reflects the manifestation of self during EC rest.

MCIN involvement (ventral anterior cingulate cortex, Table 1; BA = 24, Table 1S) in self-referential or self-awareness processing with PCIN has been reported [60], [65]–[67], constituting the cortical midline structure, the potential core of the “self”. Therefore, like T2P_L, MCIN seems to represent self-referential processing. Unlike other hubs, the MCIN hubs were only identified in the gamma band with high degree, and efficiency, which might indicate an association of the gamma frequency with self-referential mental processing mediated by the MCIN. Hubs in the gamma band are likely to reside in the vicinity of midline structures, which could be a manifestation of spectral characterizations in self-referential processes. Thus, the functional hubs we identified might be associated with the cores of the electrophysiological self, representing embedded self-referential or self-regulatory processes. In addition, because midline structures are associated with the DMN [59], the presence of MCIN as a cortical hub can be regarded as another presentation of the default state of the brain at rest.

In addition to the self-related processes corresponding to the internal world, the brain must also be sensitive to the external world during rest. In contrast to a recent study [15], our study identified cortical hubs over all frequency bands rather than within specific frequency bands. We believe that this implies that the optimized functional networks are ready for unexpected external stimuli. Because our brain networks should be able to rapidly adapt and consequently reorganize in response to sensory input, each frequency band should be prepared to adapt the brain to external stimuli. Extensive nested frequencies, in which the phase of lower frequencies modulates the amplitude of higher frequencies, have been reported [68]. The feature of nested frequencies would be one of the manifestations of an optimized multispectral brain network that would facilitate brain function in various environments. Reorganization of brain functional networks has been reported in motor tasks [69], [70], and abnormal reactivity to a task was reported with schizophrenia and focal hand dystonia, suggesting the lack of ability to adapt to the task [12], [33], [71]. Thus, the strategy for optimizing brain networks to environmental changes employs multispectral functional networks and cortical connector hubs.

In our study, hubs were primarily identified in the left hemisphere in many cases, potentially due to the influence of the handedness of the participants; it was recently reported that hemispheric asymmetry of functional connectivity depends on handedness [72]. Further studies comparing hubs with different hand preference groups are warranted.

### Methodological Considerations

The fundamental reason for the similarities or dissimilarities in the hub locations identified in our study and previous studies is a matter of debate. As we emphasized above, the first possible reason would be the difference between the EO with fixation and EC resting states. However, because even hubs in the same EO condition were not identical, other factors should be considered, such as the method of node selection (seed-based vs. whole brain segmentation) and functional connectivity estimation (power envelop correlation vs. MI). Because different methods yielded different results [5], the different methods likely represent different aspects of the brain network.

The second issue involves the absence of an individual anatomical MRI. However, MEG-MRI coregistration is possible using a template head under conditions that assure the accurate digitization of the individual head shape, with careful visual inspection [16]. We tried to follow this recommendation as much as possible. Moreover, because the nodes were based on the AAL-atlas, we minimized the possibility of placing nodes on the wrong representative brain regions.

The third issue is related to node selection. The number of source nodes used in the present study was 72, 36 for each hemisphere. There is no clear answer to the question of how many nodes would most accurately represent the brain functional network. Finer segmentation may be beneficial to obtain a more localized focus; however, the well-known field spread effect of MEG increases with increasing node number. Thus, the trade-off between finer segmentation and the field-spread effect should be considered. We supposed that our nodes were numerous enough to represent each brain region while avoiding the field spread effect. Because there is no guarantee that source-level connectivity is free from field spread effects, MI connectivity between nodes located less than 40 mm apart was discarded as a second means of ensuring minimal field spread effects [8], [35].

A final issue is the limited ability to cover the deep structures of the brain with MEG. Deep structures such as the brainstem may be key to understanding the human conscious state. Impaired neural synchronization between the brainstem and the cerebral cortex was observed in patients with lock-in-syndrome, a typical conscious state with brainstem damage [73]. Thus, despite its importance, the contribution of the brainstem to resting state functional networks is impossible to examine with MEG, as is also the case for fMRI. PET can capture brainstem activity, but it measures metabolic changes rather than direct electrical activity and consequently has a limited temporal scope. Therefore, despite a lack of coverage of deep structures, MEG is a good method to investigate the spectral characteristics of functional networks.

### Implications for Future Study and Conclusions

The examination of functional cortical hubs is as important as examining the human connectome from a neuronal perspective and will shed light on complex brain functions. Here, we showed that multispectral functional cortical hubs can be consistently identified by different hub classification measures. With this background of functional cortical hubs, the substrate of the EC resting state, diverse applications can be envisioned. Identification of the causal relationships among cortical hubs during specific tasks or brain diseases could provide new insights into brain function. Because of the dependency of cortical networks on specific tasks given to the brain, attacks to break the networks, or pathological states, examining the brain during a given task or comparing healthy and diseased brains would be useful to understand what happens in the brain. Moreover, it is even possible to examine a patient who has difficulty maintaining the EO fixation state. It would be interesting to determine which spectral responses emerge from the multispectral functional networks to adopt them to a given situation.

In conclusion, the investigation of functional networks has an impact on understanding how cortical areas form a configured structure and cooperatively interact each other. Examination of hubs in functional brain networks in multispectral ranges is possible with MEG by virtue of its high temporal resolution. We emphasized the existence of functional cortical hubs and their spatial and spectral characteristics in the EC state. Moreover, we showed their features as connector hubs that play an important role integrating functional networks across frequency bands with high degree, efficiency, and centrality.

## Supporting Information

Figure S1 **Grand averaged functional networks projected onto the cortex at each frequency band.**
(DOCX)

Table S1 **MNI coordinates and BA of each region.**
(DOCX)
