# Supplementary material for: Functional Cortical Hubs in the Eyes-Closed Resting Human Brain from an Electrophysiological Perspective Using Magnetoencephalography
Source: PLoS One. 2013 Jul 9;8(7):e68192. doi: 10.1371/journal.pone.0068192 (PMC3706585; doi:10.1371/journal.pone.0068192)

**Figure S1. Grand averaged functional networks projected onto the cortex at each frequency band (left panel: left view; middle panel: top view; right panel: right view).**

The size of each node is proportional to the degree at each location, and the thickness of each connection line between nodes is proportional to the MI value between the 2 nodes. To visualize the functional networks, MI matrices were thresholded by the top 3 percent value at each frequency band; otherwise, the network topology is difficult to recognize.


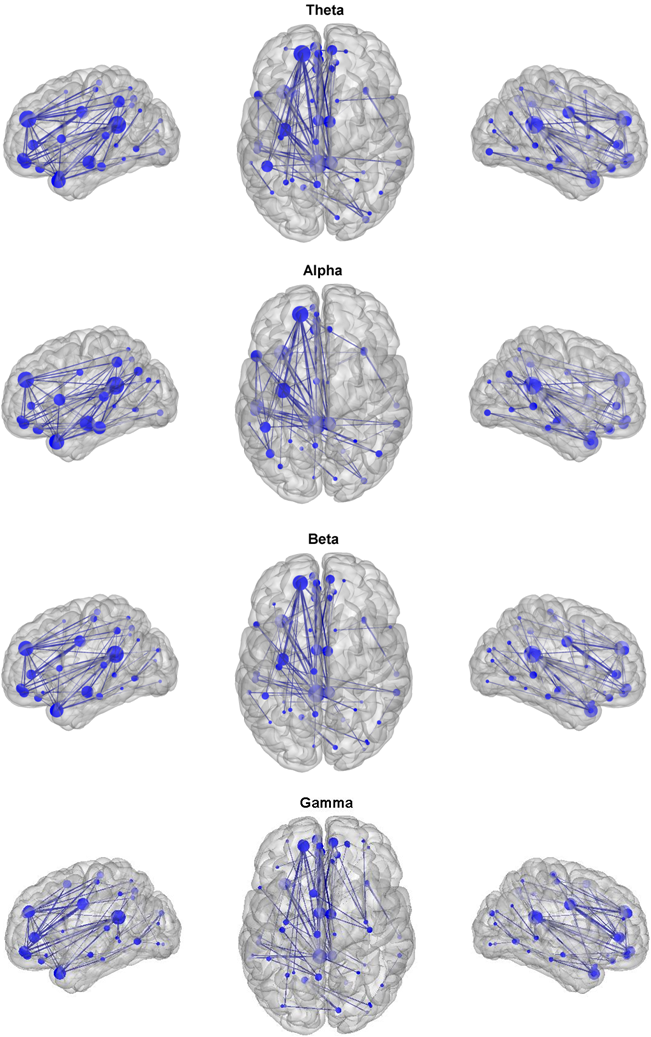

Supplement: Figure S1 — Grand averaged functional networks projected onto the cortex at each frequency band. (DOCX) [file pone.0068192.s001.docx]
