# Supplementary material for: Functional Cortical Hubs in the Eyes-Closed Resting Human Brain from an Electrophysiological Perspective Using Magnetoencephalography
Source: PLoS One. 2013 Jul 9;8(7):e68192. doi: 10.1371/journal.pone.0068192 (PMC3706585; doi:10.1371/journal.pone.0068192)
Supplement: Table S1 — MNI coordinates and BA of each region. (DOCX) [file pone.0068192.s002.docx]

**Table S1. MNI coordinates and BA of each region**

|  | Left | | | Right | | | BA |
| --- | --- | --- | --- | --- | --- | --- | --- |
| PRE | -35 | -24 | 62 | 35 | -24 | 62 | 4 |
| POST | -36 | -36 | 61 | 36 | -36 | 61 | 3 |
| F1 | -18 | 46 | 34 | 18 | 46 | 34 | 9 |
| F2 | -34 | 45 | 26 | 34 | 45 | 26 | 10 |
| F3OP | -53 | 13 | 11 | 54 | 11 | 13 | 44 |
| F3T | -54 | 11 | 23 | 54 | 12 | 23 | 45 |
| F1M | -5 | 46 | 32 | 6 | 49 | 32 | 9 |
| SMA | -10 | 6 | 61 | 10 | 6 | 61 | 6 |
| PCL | -7 | -28 | 69 | 7 | -28 | 69 |  |
| F1O | -19 | 50 | -14 | 17 | 50 | -14 | 11 |
| F1MO | -7 | 53 | -9 | 7 | 53 | -9 | 11 |
| F2O | -31 | 52 | -10 | 30 | 52 | -10 |  |
| F3O | -39 | 31 | -15 | 39 | 31 | -15 | 47 |
| GR | -6 | 36 | -21 | 5 | 36 | -21 |  |
| T1 | -59 | -42 | 1 | 59 | -42 | 1 | 22 |
| HES | -54 | -32 | 12 | 56 | -32 | 12 | 41&42 |
| T2 | -61 | -44 | -3 | 61 | -44 | -3 | 21 |
| T3 | -53 | -26 | -23 | 59 | -26 | -23 | 20 |
| P1 | -26 | -59 | 61 | 26 | -59 | 61 | 7 |
| P2 | -31 | -60 | 43 | 31 | -60 | 43 | 7 |
| AG | -42 | -68 | 37 | 45 | -68 | 34 | 39 |
| SMG | -46 | -47 | 48 | 46 | -47 | 48 | 40 |
| PQ | -6 | -62 | 50 | 8 | -62 | 47 | 7 |
| O1 | -34 | -78 | 27 | 35 | -83 | 27 | 19 |
| O2 | -35 | -84 | 14 | 36 | -84 | 14 | 19 |
| O3 | -33 | -83 | -8 | 33 | -88 | -9 | 18 |
| Q | -7 | -87 | 25 | 10 | -88 | 25 | 19 |
| V1 | -10 | -74 | 8 | 10 | -74 | 8 | 23 |
| LING | -17 | -62 | -4 | 16 | -64 | -4 | 19 |
| FUSI | -31 | -49 | -15 | 31 | -49 | -15 | 37 |
| T1P | -26 | 17 | -31 | 33 | 19 | -28 | 38 |
| T2P | -33 | 18 | -37 | 33 | 18 | -37 | 38 |
| ACIN | -5 | 42 | 6 | 9 | 40 | 6 | 32 |
| MCIN | -5 | -9 | 39 | 4 | -10 | 38 | 24 |
| PCIN | -5 | -44 | 23 | 5 | -43 | 23 | 23 |
| HIP | -32 | -14 | -17 | 32 | -14 | -17 |  |
